# Biomarkers in Ewing Sarcoma: The Promise and Challenge of Personalized Medicine. A Report from the Children’s Oncology Group

**DOI:** 10.3389/fonc.2013.00141

**Published:** 2013-06-06

**Authors:** Neerav Shukla, Joshua D. Schiffman, Damon Reed, Ian J. Davis, Richard B. Womer, Stephen L. Lessnick, Elizabeth R. Lawlor, The COG Ewing Sarcoma Biology Committee

**Affiliations:** ^1^Department of Pediatrics, Memorial Sloan-Kettering Cancer Center, New York, NY, USA; ^2^Division of Pediatric Hematology/Oncology, Department of Oncological Sciences, Center for Children’s Cancer Research, Huntsman Cancer Institute, The University of Utah, Salt Lake City, UT, USA; ^3^Department of Sarcoma Oncology, Moffitt Cancer Center, Tampa, FL, USA; ^4^Lineberger Comprehensive Cancer Center, University of North Carolina, Chapel Hill, NC, USA; ^5^Children’s Hospital of Philadelphia, University of Pennsylvania, Philadelphia, PA, USA; ^6^Department of Pediatrics, University of Michigan, Ann Arbor, MI, USA; ^7^Department of Pathology, University of Michigan, Ann Arbor, MI, USA

**Keywords:** Ewing sarcoma, biomarkers, prognostic, predictive

## Abstract

A goal of the COG Ewing Sarcoma (ES) Biology Committee is enabling identification of reliable biomarkers that can predict treatment response and outcome through the use of prospectively collected tissues and correlative studies in concert with COG therapeutic studies. In this report, we aim to provide a concise review of the most well-characterized prognostic biomarkers in ES, and to provide recommendations concerning design and implementation of future biomarker studies. Of particular interest and potentially high clinical relevance are studies of cell-cycle proteins, sub-clinical disease, and copy number alterations. We discuss findings of particular interest from recent biomarker studies and examine factors important to the success of identifying and validating clinically relevant biomarkers in ES. A number of promising biomarkers have demonstrated prognostic significance in numerous retrospective studies and now need to be validated prospectively in larger cohorts of equivalently treated patients. The eventual goal of refining the discovery and use of clinically relevant biomarkers is the development of patient specific ES therapeutic modalities.

## Introduction

First described by James Ewing as an endothelioma of bone (Ewing, 1972), Ewing sarcoma (ES) was for many years primarily a histologic diagnosis of exclusion based on its highly undifferentiated, small round blue cell phenotype. Although ES presents most commonly in bones in the pediatric and adolescent populations, in older patients it is predominantly a soft tissue tumor (Karski et al., 2013). Historically, tumors in specific anatomic locations or with features suggestive of differentiation resulted in distinct diagnoses such as Askin tumors, peripheral primitive neuroectodermal tumor (PNET), or extraosseous ES. However, based on the identification of a common genetic lesion, and similar clinical behavior and response to treatment, the World Health Organization now collectively refers to these tumors as ES (Fletcher et al., 2013).

It was the advent of consistency in diagnosis that enabled cooperative groups worldwide to develop multi-center ES clinical trials. Over the past three decades these trials have systematically evaluated and optimized local and systemic treatment protocols for patients with ES (Rosen et al., 1974; Miser et al., 1987; Nesbit et al., 1990; Kung et al., 1993; Craft et al., 1998; Ferrari et al., 1998; Paulussen et al., 1998; Saylors et al., 2001). The current standard of care for North American pediatric cooperative group patients with localized ES was derived from two recent Phase III clinical trials from the Children’s Oncology Group (COG) (Grier et al., 2003; Womer et al., 2012). Patients with non-metastatic disease receive multi-agent chemotherapy every 2 weeks as neoadjuvant therapy before local control, which comprises surgery, radiation or both, and then adjuvant therapy for an additional several cycles. With this aggressive regimen, patients with localized disease have event free survival (EFS) rates of around 75%. Unfortunately, approximately 20–30% of patients present with metastases, and these patients have drastically poorer outcomes since systemic chemotherapy trials have not improved durable remission rates for patients with metastatic ES (Cotterill et al., 2000; Rodriguez-Galindo et al., 2008).

Outside of metastasis there is a large body of literature that supports other clinical-pathologic features as markers of high-risk disease. Increasing tumor size, decreased tumor necrosis after neoadjuvant chemotherapy, central tumor site (axial versus appendicular), and increasing patient age have all been implicated as negative prognostic features. None of these are as significant as the presence of metastatic disease and studies have demonstrated variability in these individual features (Sauer et al., 1987; Cotterill et al., 2000; Oberlin et al., 2001; Paulussen et al., 2001; Martin and Brennan, 2003; Bacci et al., 2004; Lin et al., 2007; Rodriguez-Galindo et al., 2008; Lee et al., 2010). Thus, current North American cooperative group therapeutic ES trials stratify patients based solely on the presence or absence of metastases. Furthermore, we have little insight into which patients with localized disease are at risk for recurrence or which patients with metastatic disease are curable with conventional therapy. It would be very beneficial if practitioners could predict which patients are unlikely to be cured by standard therapy so that they can be considered for treatment with novel agents and regimens. As new agents are introduced into practice it will also be important to introduce them rationally, prescribing them to optimal patient cohorts who will be most likely to respond.

In an effort to advance knowledge of tumor biology and treatment response in pediatric cancer patients the COG has established disease-specific biology committees. The COG Ewing’s Biology Committee consists of physicians and researchers with expertise in ES biology, pre-clinical, and translational research, and clinical care. One of the goals of the committee is to enable the identification of reliable biomarkers that can predict treatment response and outcome through the use of prospectively collected tissues and correlative studies in concert with COG therapeutic studies. This report aims to provide a concise review of the most well-characterized prognostic biomarkers in ES, and to provide recommendations concerning design and implementation of future biomarker studies.

## Biomarkers and REMARK Criteria

In the current era of individualized therapies and the goal of “personalized medicine,” the term biomarker is increasingly en vogue. However, attention to the precise definitions of a biomarker, and how the biomarker was developed, validated, and applied to clinical protocols is critical. The National Institutes of Health defines a *biomarker* as a characteristic that is objectively measured and evaluated as an indicator of normal biologic processes, pathogenic processes, or pharmacologic responses to a therapeutic intervention (De Gruttola et al., 2001). The characteristics of a useful biomarker include the following: provide a clear risk/benefit ratio to facilitate clinical decisions, available in an efficient, and cost-effective manner, can be assessed on easily obtainable samples, and able to be performed on available technological platforms (Hodgson et al., 2009).

Biomarkers can be subdivided into two types: prognostic and predictive. The majority of biomarkers studied in ES are *prognostic*. Prognostic biomarkers provide information about the outcome of a disease following standard therapy (La Thangue and Kerr, 2011). As discussed above, the presence of metastatic disease at diagnosis is currently the most clinically informative prognostic biomarker in ES. Based on knowledge of this feature (i.e., metastasis), current protocols may augment therapy and/or add novel agents to patients with metastatic disease in an attempt to improve outcomes. In comparison, *predictive* biomarkers provide information about the likelihood of response to a certain therapeutic modality, such as a novel agent. This group of predictive biomarkers allows for a more individualized approach to treatment, as it provides direct information linking drug and tumor response (La Thangue and Kerr, 2011). To date, these types of biomarkers are lacking in ES.

Laboratory advances and improvements in tumor banking, have led to a dramatic increase in studies exploring the use of biomarkers. However, conflicting results from studies analyzing the same biomarker often emerge. Contradictory findings may arise from issues such as methodological differences, poor study designs, non-standardized assays, and small sample sizes (McShane et al., 2005). To address these issues, level of evidence (LOE) scales for tumor marker studies were established by the American Society of Clinical Oncology (Table 1), and these LOE scales continue to be reevaluated and modified (Hayes et al., 1996; Simon et al., 2009). When designed properly, prospective studies provide the most reliable data for biomarker analysis with little to no additional validation necessary. Significant efforts to optimize the reporting of biomarker studies have also been recently made. The National Cancer Institute published Reporting Recommendations for Tumor Marker Prognostic Studies (REMARK) guidelines in 2005 and updated these in 2012 (McShane et al., 2005; Altman et al., 2012) (Table 2). These guidelines require that for a biomarker study to be considered adequate, it must: (1) clearly describe treatment modalities of all patients, (2) utilize reproducible methodology, and (3) contain a well-defined and robust biostatistical plan. In pediatric oncology, where prospective clinical trials take many years to complete and require the participation of clinicians at numerous institutions, it is imperative that prospective biomarker studies be fastidiously designed in order to ensure that the accumulated data is adequate and interpretable.

**Table 1:** **Levels of evidence for grading clinical utility of tumor markers**.

Level	Type of evidence
I	Evidence from a single, high-powered, prospective, controlled study that is specifically designed to test marker or evidence from meta-analysis and/or overview of level II or III studies. In the former case, the study must be designed so that therapy and follow-up are dictated by protocol. Ideally, the study is a prospective, controlled randomized trial in which diagnostic and/or therapeutic clinical decisions in one arm are determined at least in part on the basis of marker results, and diagnostic and/or therapeutic clinical decisions in the control arm are made independently of marker results. However, study design may also include prospective but not randomized trials with marker data and clinical outcome as primary objective.
II	Evidence from study in which marker data are determined in relationship to prospective therapeutic trial that is performed to test therapeutic hypothesis but not specifically designed to test marker utility (i.e., marker study is secondary objective of protocol). However, specimen collection for marker study and statistical analysis are prospectively determined in protocol as secondary objectives.
III	Evidence from large but retrospective studies from which variable numbers of samples are available or selected. Therapeutic aspects and follow-up of patient population may or may not have been prospectively dictated. Statistical analysis for tumor marker was not dictated prospectively at time of therapeutic trial design.
IV	Evidence from small retrospective studies that do not have prospectively dictated therapy, follow-up, specimen selection, or statistical analysis. Study design may use matched case?controls, etc.
V	Evidence from small pilot studies designed to determine or estimate distribution of marker levels in sample population. Study design may include “correlation” with other known or investigational markers of outcome but is not designed to determine clinical utility.

Reprinted by permission from Oxford University Press: Journal of the National Cancer Institute (Hayes et al., 1996).

**Table 2:** **Reporting recommendations for tumor marker prognostic studies**.

Guidelines for the REporting of tumor MARKer studies (REMARK)
**Introduction**
State the marker examined, the study objectives, and any prespecified hypotheses
**Materials and methods**
Patients
Describe the characteristics (e.g., disease stage or comorbidities) of the study patients, including their source and inclusion and exclusion criteria
Describe treatments received and how chosen (e.g., randomized or rule-based)
Specimen characteristics
Describe the type of biological material used (including control samples) and methods of preservation and storage
Assay methods
Specify the assay method used and provide (or reference) a detailed protocol, including specific reagents or kits used, quality control procedures, reproducibility assessments, quantitation methods, and scoring and reporting protocols. Specify whether and how assays were performed blinded to the study end point
Study design
State the method of case selection, including whether the study design was prospective or retrospective and whether stratification or matching (e.g., by stage of disease or age) was used. Specify the time period from which cases were taken, the end of the follow-up period, and the median follow-up time
Precisely define all clinical end points examined
List all candidate variables initially examined or considered for inclusion in models
Give rationale for sample size; if the study was designed to detect a specified effect size, give the target power and effect size
Statistical analysis methods
Specify all statistical methods, including details of any variable selection procedures and other model-building issues, how model assumptions were verified, and how missing data were handled
Clarify how marker values were handled in the analyses; if relevant, describe methods used for cutpoint determination
**Results**
Data
Describe the flow of patients through the study, including the number of patients included in each stage of the analysis (a diagram may be helpful) and reasons for dropout. Specifically, both overall and for each subgroup extensively examined report the numbers of patients and the number of events
Report distributions of basic demographic characteristics (at least age and sex), standard (disease-specific) prognostic variables, and tumor marker, including numbers of missing values
Analysis and presentation
Show the relation of the marker to standard prognostic variables
Present univariate analyses showing the relation between the marker and outcome, with the estimated effect (e.g., hazard ratio and survival probability). Preferably provide similar analyses for all other variables being analyzed. For the effect of a tumor marker on a time-to-event outcome, a Kaplan–Meier plot is recommended
For key multivariable analyses, report estimated effects (e.g., hazard ratio) with confidence intervals for the marker and, at least for the final model, all other variables in the model
Among reported results, provide estimated effects with confidence intervals from an analysis in which the marker and standard prognostic variables are included, regardless of their statistical significance
If done, report results of further investigations, such as checking assumptions, sensitivity analyses, and internal validation
**Discussion**
Interpret the results in the context of the prespecified hypotheses and other relevant studies; include a discussion of limitations of the study
Discuss implications for future research and clinical value

Reprinted by permission from American Society of Clinical Oncology: The Journal of Clinical Oncology (McShane et al., 2005).

## Prognostic Biomarkers in ES

Numerous studies of prognostic biomarkers in ES as well as several comprehensive reviews of these biomarkers have recently been published (Pinto et al., 2011; van Maldegem et al., 2012; Wagner et al., 2012). This review focuses on four main categories: *EWSR1* translocation type, cell-cycle proteins, copy number alterations (CNAs), and sub-clinical disease measurement. Fusion type will be discussed to demonstrate the importance of prospective evaluation and validation of biomarkers in the context of evolving therapy. The remaining categories were selected for in depth discussion after consideration of REMARK criteria. In the following sections we will highlight the features of each of these putative biomarkers that lead us to propose that their parallel evaluation and validation in the next series of prospective therapeutic trials is warranted. Several of these, including CNAs and cell-cycle proteins were recently discussed at a European Network for Cancer Research in Children and Adolescents (ENCCA) summit of 35 international experts (Kovar et al., 2012). In addition, several additional “emerging” biomarkers of potential prognostic significance were discussed at the ENCCA summit and the reader is directed to the published summary of these discussions for more detailed information (Kovar et al., 2012).

### *EWSR1* translocation type

The molecular hallmark of ES is a recurrent chromosomal translocation involving the *EWSR1* gene and one of several different genes belonging to the *ETS* family (Delattre et al., 1992). In approximately 85% of these translocations, the 5′ portion of the *EWSR1* gene on chromosome 22 is fused to the 3′ portion of the *FLI1* gene on chromosome 11. The most common fusion type joins exon 7 of *EWSR1* with exon 6 of *FLI1*, also known as the type-1 fusion. However, numerous less common breakpoints between the two genes have been identified. Furthermore, about 10% of cases involve alternate *ETS* family genes as the 3′ translocation partner. A detailed review of the various fusion types described in ES was recently published by Sankar and Lessnick (2011).

Associations between fusion type and prognosis were observed in the late 1990s through studies of archival tumors and outcome data. de Alava et al. (1998) analyzed 99 patient samples and found that patients with tumors harboring a type-1 fusion had a significantly better overall survival compared to those with other fusion types. The difference was observed when all patients were analyzed, as well among those patients who presented with localized disease. Similarly, Zoubek et al. (1996) performed a retrospective analysis of 85 tumor samples from patients enrolled on the European Cooperative ES Studies. In this study a significant reduction in relapse rate was observed in patients with localized disease whose tumors harbored a type-1 fusion.

In an attempt to validate these retrospective studies on prospectively collected sets of tumors from equivalently treated patients, both COG and Euro-Ewing evaluated fusion status and outcomes in patients diagnosed between 1999 and 2007. Strikingly, these studies failed to confirm the original findings. Reporting on 578 patients enrolled on the European EURO-E.W.I.N.G. 99 trial, Le Deley et al. (2010) failed to observe an impact of fusion type on risk of progression or relapse. Likewise, van Doorninck et al. studied 119 prospectively collected patient samples from two consecutive COG trials and again failed to identify differences in clinical outcomes based on *EWSR1* fusion status. While the original finding of fusion type as a prognostic biomarker may have been due to the bias of retrospective studies, it is possible that the increased intensity of current treatment regimens eliminated the impact of *EWSR1* fusion type on clinical outcome (Barr and Meyer, 2010; van Doorninck et al., 2010).

Although variations in *EWSR1* fusion partner can no longer be considered prognostic, recent discoveries have complicated the clinical scenario. There have been several recent reports of novel non-*EWSR1* fusions in tumors with Ewing-like features (CIC-DUX and BCOR-CCND fusions) (Italiano et al., 2012; Pierron et al., 2012). In the absence of data to support an alternate diagnosis or approach to treatment, these patients are treated according to ES standard care or are enrolled on ES therapeutic trials. Based on their rarity, unless outcomes for these tumors prove to be dramatically different from more classical ES, it is statistically improbable that studies of these cases will ever meet REMARK criteria for definitive designation as prognostic biomarkers. Ideally, a better understanding of the biologic heterogeneity of ES may offer mechanistic insights that ultimately direct optimal clinical care for these variant cases.

In summary, current levels of evidence strongly suggest that among the greater than 90% of ES tumors that harbor *EWSR1* rearrangements, fusion type is no longer a reliable prognostic marker and should not be used to stratify therapy or instruct treatment decisions.

### Cell-cycle proteins

The cell-cycle pathway and its multiple protein components are frequently altered in cancer. In ES, genetic alterations affecting the pRB-dependent cell-cycle regulation pathway have been described including deletions of both *CDKN2A (INK4A/ARF)* and *RB1*. Kovar et al. (1997) first described *CDKN2A* deletions in 30% of tumors (*N* = 8/27) and 52% of ES cell lines (*N* = 12/23) and several retrospective studies have demonstrated an association between *CDKN2A* alterations and clinical outcome in ES patients. Wei et al. (2000) identified *CDKN2A* deletions in 18% of analyzed tumor samples (*N* = 7/39), while Tsuchiya et al. (2000) found *CDKN2A* deletions in 17% of tumor samples (*N* = 4/24). Patients in both studies were found to have worse disease-specific survival in univariate and multivariate analyses. Maitra et al. (2001) identified *CDKN2A* downregulation by immunohistochemistry in 20% of patients (*N* = 4/20), and this correlated with metastatic disease at presentation and trended toward shortened survival. A meta-analysis examining the prognostic significance of *CDKN2A* alterations in ES based on six separate studies (*N* = 188) concluded that the estimated pooled risk ratio (RR) for worse outcome with *CDKN2A* alterations was 2.17 [95% confidence interval (95% CI), 1.55–3.03; *P* < 0.001] and the estimated pooled RR of metastasis at diagnosis was 2.60 (*N* = 164 eligible, 95% CI, 1.71–3.97; *P* < 0.001) (Honoki et al., 2007). Finally, using multiplex ligation-dependent probe amplification (MLPA), homozygous deletion of *CDKN2A* was identified in 44% of cell lines (*N* = 4/9) and 10% of primary tumors (*N* = 4/42) (Brownhill et al., 2007). Hemizygous deletion was detected in an additional 22 and 5% of samples, respectively. In contrast to previous reports, this study did not identify prognostic value of *CDKN2A* deletions or protein expression. However, given that only 4 patients with *CDKN2A* were identified in this study, it is difficult to draw definitive conclusions. Based on the cumulative data, it is the opinion of this committee that the evidence to support *CDKN2A* loss as a negative prognostic marker is strong, and worthy of prospective validation.

The potential of *TP53* mutational status as a prognostic biomarker in ES also has been evaluated in retrospective studies. Using immunohistochemistry, Abudu et al. (1999) detected TP53 over-expression indicative of non-functional protein in 14% of tumor samples (*N* = 7/52) and this over-expression was associated with advanced disease at diagnosis, poorer treatment response, and a worse overall survival. Significantly, this effect was independent of site, local treatment, or tumor necrosis. Similarly, a study by de Alava et al. (2000) identified TP53 over-expression based on immunoreactivity in 11% of tumor samples (*N* = 6/55) and increased p53 protein expression was found to be the strongest prognostic factor that was associated with worse overall survival. Huang et al. (2005) reported *TP53* mutations in 13.3% of patient samples (*N* = 8/60), as well as *CDKN2A* homozygous deletions in another 13.3% of samples (*N* = 8/60). *TP53* mutations and/or *CDKN2A* deletions were significantly associated with a poor response to chemotherapy (*P* < 0.0001) and, in a multivariate analysis, *TP53* and/or *CDKN2A* alteration status as a single combined variable was identified as the most significant prognostic factor (*P* < 0.001). Finally, using immunohistochemistry and fluorescent *in situ* hybridization (FISH), Lopez-Guerrero et al. (2011) analyzed cell-cycle regulation markers in 324 cases of ES. They reported a significant association between increased TP53 expression and metastatic disease (*P* = 0.025), and worse progression-free survival (*P* = 0.012) and disease-specific survival (*P* = 0.006) in patients with localized disease.

In summary, compelling data from several retrospective studies implicates alterations of *TP53* and *CDKN2A* as negative prognostic biomarkers in ES. Currently, COG and the ES Biology Committee are performing a large-scale analysis of *TP53* and *CDKN2A* status in over 150 prospectively collected tumors from patients treated on the most recent AEWS0031 therapeutic study. Should this study confirm prior observations, analysis of these cell-cycle regulatory proteins will become a strong candidate for inclusion as a prognostic biomarker that can inform treatment decisions in future clinical trials.

### Copy number alterations

Genomic instability with subsequent CNAs have been well-documented in ES and these alterations have been recently reviewed by Jahromi et al. (2011). The recurrent CNAs most commonly described to be associated with outcome are summarized in Table 3 along with reference to the primary manuscripts. The most commonly reported CNAs in ES are trisomy of chromosome 8, trisomy of chromosome 12, and gain of chromosome 1q. The technology to measure these CNAs has improved throughout the years, and likewise so has the ability to detect and correlate CNA with clinical outcome. Using a variety of platforms, several recurring regions of gains and losses with clinical relevance have been described. However, these retrospective studies use different approaches to identify CNAs among varying number of patients leading to different trends and degrees of association. A prospective analysis of CNAs and clinical outcome has not yet been undertaken.

**Table 3:** **Recurrent CNAs and outcome correlations in Ewing sarcoma studies**.

Region	Technology	Total with CNA	EFS (%)	Significance	OS (%)	Significance	Study
1p36.3 loss	Cytogenetics and FISH	9/51 (18%)	17 vs. 81	*P* = 0.004			Hattinger et al. (1999)
1q21-q22 gain	CGH	5/20 (25%)	–	–	50 vs. 78	*P* = 0.57 (trend)	Armengol et al. (1997)
	G-banded karyotype	3/20 (15%)	–	–	0 vs. 61	NA	Kullendorff et al. (1999)
	CGH	5/28 (18%)	40 vs. 59	*P* = 0.30 (trend)	40 vs. 60	*P* = 0.45 (trend)	Tarkkanen et al. (1999)
	CGH	21/67 (31%)	–	–	41 vs. 87*	*P* = 0.32 (trend, multivariate)	Mackintosh et al. (2012)
6p21.1 gain	CGH	3/28 (11%)	0 vs. 63	***P* = 0.04**	0 vs. 64	***P* = 0.004**	Tarkkanen et al. (1999)
8 gain	CGH	7/20 (35%)	–	–	50 vs. 84	*P* = 0.16 (trend)	Armengol et al. (1997)
	CGH	10/28 (36%)	40 vs. 65	*P* = 0.16 (trend)	45 vs. 63	*P* = 0.39 (trend)	Tarkkanen et al. (1999)
	Cytogenetics and FISH	10/21 (48%)	90 vs. 60	*P* = 0.1528 (trend)	–	–	Zielenska et al. (2001)
	SNP Microarray (MIP)	15/40 (38%)	35 vs. 80	***P* = 0.0059**	26 vs. 100	***P* = 0.00038**	Jahromi et al. (2012)
12 gain	CGH	5/20 (25%)			50 vs. 78	*P* = 0.30 (trend)	Armengol et al. (1997)
	CGH	3/28 (11%)	33 vs. 59	*P* = 0.36 (trend)	67 vs. 55	*P* = 0.67 (trend)	Tarkkanen et al. (1999)
	Cytogenetics and FISH	6/16 (38%)	50 vs. 94	*P* = 0.0751 (trend)			Zielenska et al. (2001)
16q loss	CGH	11/52 (21%)	–	–	NA	***P* = 0.0006**	Ozaki et al. (2001)
	SNP Microarray (MIP)	4/40 (10%)	25 vs. 70	*P* = 0.11 (trend)	50 vs. 74	*P* = 0.26 (trend)	Jahromi et al. (2012)
20 gain	Cytogenetics	10/75 (13%)	16 vs. 57	***P* = 0.006**	30 vs. 59	***P* = 0.008**	Roberts et al. (2008)
	SNP Microarray (MIP)	7/40 (18%)	30 vs. 68	***P* = 0.012**	0 vs. 79	***P* = 0.00013**	Jahromi et al. (2012)
Complex	*G*-banded karyotype	3/20 (15%)**	–	–	0 vs. 61	NA	Kullendorff et al. (1999)
	Cytogenetics and FISH	9/22 (41%)***	44 vs. 100	***P* = 0.034**	–	–	Zielenska et al. (2001)
	CGH	13/48 (27%)****	–	–	15 vs. 50	***P* = 0.009**	Ozaki et al. (2001)
	CGH	12/25 (48%)*****	–	–	25 vs. 80	***P* = 0.034** (multivariate)	Ferreira et al. (2008)
	Cytogenetics	22/75 (29%)**	29 vs. 50	*P* = 0.08 (trend)	47 vs. 58	***P* = 0.05**	Roberts et al. (2008)
	CGH	11/23 (48%)*****	20 vs. 42	***P* = 0.049**	30 vs. 67	***P* = 0.030**	Savola et al. (2009)
	SNP Microarray (MIP)	20/40 (50%)*****	58 vs. 68	*P* = 0.48 (discrete) ***P* = 0.017** (continuous)	52 vs. 93	***P* = 0.027** (discrete) ***P* = 0.00005** (continuous)	Jahromi et al. (2012)
Multifactor Copy Number (MCN)-index	SNP Microarray (MIP)	*N* = 19/40 (48%)******	40 vs. 83	***P* = 0.013**	39 vs. 100	***P* = 0.00013**	Jahromi et al. (2012)

*Cumulative Survival.

**>50 chromosomes.

***≥1 structurally rearranged chromosomes.

****≥5 CNAs.

*****>3 CNAs.

******MCN-index: ≥1 CNA in 20q13.2 gain, 20q13.13 gain, MYC gain, 16q24.1 loss, 16q23.3-24.1 loss, Trisomy 5, Trisomy 8, Trisomy 20.

NA = Not analyzed.

MIP = Molecular Inversion Probe.

The bold font represent significant studies with p-value less than or equal to 0.05.

In summary, independent studies of both small and large tumor cohorts have identified individual and global patterns of CNAs as putative prognostic biomarkers in ES. We anticipate that the continued improvement in next generation sequencing platforms will allow for greater characterization of structural variations in tumors, and will generate even more data to test associations between CNAs and clinical outcome. It is the recommendation of this committee that tumor and germline DNA be collected from all patients registered on future therapeutic studies of ES in order that CNAs and other genetic mutations can be evaluated as prognostic and predictive biomarkers in homogeneously treated patients. To that end, COG has discussed the prospective incorporation of CNA and genomic analysis in their upcoming ES trial for relapsed/refractory patients.

### Sub-clinical disease

Assessment of minimal residual disease (MRD) has been established as a critical part of therapeutic decision making in childhood acute lymphoblastic leukemia (Biondi et al., 2000; Borowitz et al., 2008). Standardized methodologies and MRD assessment time points have been incorporated into COG and other cooperative group lymphoblastic leukemia protocols, and serve as prognostic biomarkers for patient risk stratification. As detailed below, attempts to validate methodologies and prognostic correlations for sub-clinical disease detection in ES have primarily used reverse-transcriptase polymerase chain reaction (RT-PCR) and flow cytometry.

#### RT-PCR

RT-PCR assays for sub-clinical disease are designed to identify pathognomonic ES related fusion transcripts in blood and/or bone marrow as evidence of occult micrometastatic disease or persistent disease following systemic therapy. Through serial dilution experiments of established ES cell lines, this methodology was proven to have sufficient sensitivity to detect a single tumor cell among 10^6^ normal cells (Peter et al., 1995; Pfleiderer et al., 1995; West et al., 1997). The largest published study examined *EWSR1-FLI1* and *EWSR1-ERG* transcript levels in the bone marrow and peripheral blood taken at the time of diagnosis of ES in 172 patients, 140 of whom were enrolled on French Society of Pediatric Oncology (SFOP) protocols and therefore received similar therapy (Schleiermacher et al., 2003). RT-PCR positive bone marrow samples were identified in 27% of evaluated patients (*N* = 36/131), and 19% of patients (*N* = 18/92) with non-metastatic disease at presentation. Circulating transcripts were identified in 20% of patients (*N* = 29/144) at diagnosis, and were more frequently observed in patients with large tumor burdens. In patients with localized disease, RT-PCR positivity in bone marrow and peripheral blood correlated with significantly poorer outcomes. In contrast, a study of peripheral blood samples from 26 children was unable to identify a significant progression-free survival difference in patients with detectable fusion transcript at diagnosis (Avigad et al., 2004). However, this study suggested that identification of circulating transcript during disease follow-up was predictive of recurrence. Finally, Zoubek et al. (1998) examined bone marrow samples from 35 newly diagnosed patients. Transcript was detected in 30% of patients (*N* = 7/23) with localized disease, 50% of patients (*N* = 3/6) with isolated pulmonary metastases, and 100% of patients (*N* = 6/6) with bone metastases. However, the study did not establish a correlation between marrow positivity for ES transcript and progression-free disease. Results of other smaller studies have been recently summarized by Wagner et al. (2012).

To rigorously address whether the detection of circulating tumor transcript is of prognostic significance, the multi-center European EURO-E.W.I.N.G. 99 trial prospectively collected bone marrow samples for over 10 years. As the first large prospective trial examining sub-clinical disease via RT-PCR in ES patients, the findings of this study will be critical to evaluate the feasibility and usefulness of this modality as a biomarker for ES. Based on our own experience with a much smaller cohort of patients in COG we, as a committee, are skeptical that RT-PCR-based assays will be clinically optimal for prognostication and treatment stratification. We base this assertion on our combined observations regarding issues of technical reproducibility of the assay between individual laboratories, and the technical expertise required to consistently obtain sufficient quality RNA for valid and reliable RT-PCR analysis. Although these issues could be addressed with the establishment of a central College of American Pathologists (CAP)-Clinical Laboratory Improvement Act (CLIA)-certified reference laboratory, the issue of RNA degradation in sample shipments would remain. In addition, RT-PCR-based analysis requires knowledge of the precise breakpoint. With the increasing use at many COG institutions of closed needle biopsy for diagnostic tissue collection and fluorescence *in situ* hybridization (FISH) for molecular diagnosis, isolation of quality RNA has become less practical. Feasibility will only diminish as additional rare non-*EWSR1* translocations are identified.

In summary, although of potential prognostic significance, technical and logistic realities regarding tissue collection and RNA-based studies of blood and bone marrow specimens significantly diminish this committee’s enthusiasm for RT-PCR analysis of sub-clinical disease in routine clinical practice. Should the aforementioned Euro-Ewing study validate RT-PCR of bone marrow as a significant prognostic variable, this issue will need to be re-addressed. At such time, consideration would need to be given to optimizing collection and submission of quality RNA and to creation of a CAP-CLIA certified COG reference laboratory.

#### Flow cytometry

Recently, flow cytometric approaches have been used to identify sub-clinical disease in ES (Dubois et al., 2010; Ash et al., 2011). These assays use the cell-surface glycoprotein CD99 to identify tumor cells. Dubois et al. (2010) used a gating strategy to identify CD99+/CD45− cells in normal peripheral blood samples spiked with varying titrations of cultured ES cells. They were able to identify one tumor cell among 5 × 10^5^ peripheral blood mononuclear cells. Ash et al. (2011) used a similar gating strategy and identified ES cells that were CD99+/CD90+/CD45−. Diagnostic bone marrow samples from 46 ES patients, including 35 with localized disease, were examined. Tumor cells were identified in all 46 diagnostic marrow samples, ranging from 0.001–0.4% positivity. Ten control marrow samples from patients without malignancies were all negative. Furthermore, they identified high CD56 expression on the tumor cells as a significant poor prognostic factor.

Flow cytometric based platforms have several practical advantages over RT-PCR. Flow cytometric assays require significantly less labor, and are easier to standardize across different centers. For central laboratories, sample shipment does not carry the same degree of concern about degradation as RNA-based assays. Finally, unlike RT-PCR in which knowledge of the precise fusion type is required, a single flow cytometric assay could potentially be used for all patients.

These two initial studies of flow cytometry for sub-clinical disease detection confirm feasibility of the approach and provide preliminary support for the potential prognostic significance of circulating tumor cells. Studies are now underway to validate these findings within the context of current and planned COG studies. Specifically, bone marrow samples are being prospectively analyzed from newly diagnosed patients through the AEWS07B1 banking study and on patients with recurrent disease through both AEWS07B1 and through ADVL1221.

### Other studies

Many other prognostic markers in ES have been studied and associated with significant differences in outcome (Table 4). Unfortunately, the reporting standards of most of these studies do not fulfill REMARK criteria, with treatment variability and inadequate sample size being frequent problems. Further validation of the most promising of these studies is essential. As a first step, retrospective analysis of larger cohorts of prospectively collected and banked tumor tissues should be used to validate early findings in independent patient cohorts. Putative biomarkers that hold up to expanded retrospective-prospective analysis could then be considered for inclusion and validation in parallel with future therapeutic trials. Ideally, biomarkers that advance to prospective analysis will be measureable by accessible and straightforward assays that are amenable to evaluation at multiple, non-specialized sites. For example assays that require immunohistochemistry of fixed tumor specimens or analysis of peripheral blood would be preferred to those that require significant technical expertise or fresh tissue.

**Table 4:** **Recent studies examining potential Ewing sarcoma biomarkers**.

Study	Methodology	Findings	*P*-value
Ohali et al. (2003)	Analysis of telomerase activity in post-therapy peripheral blood samples of 26 patients	High telomerase activity is correlated with poorer PFS	*P* < 0.0001
Fuchs et al. (2004)	Immunohistochemical analysis of vascular endothelial growth factor (VEGF) expression in 31 diagnostic tumor samples	Positive VEGF expression is correlated with poorer OS	*P* = 0.0047
Kreuter et al. (2006)	Immunohistochemical analysis of vascular endothelial growth factor-A (VEGF-A) expression in 40 diagnostic tumor samples	Positive VEGF-A expression is correlated with improved OS	*P* = 0.013
Cheung et al. (2007)	Quantitative RT-PCR analysis of six-transmembrane epithelial antigen of the prostate 1 (*STEAP1)*, cyclin D1 (*CCND1)*, and NKX2-2 transcription factor (*NKX2-2)* in 35 histologically normal diagnostic bone marrow samples	Increased marrow expression of *STEAP1*, *CCND1*or *NKX2-2* is correlated with poorer OS	*P* = 0.0001
Yabe et al. (2008)	Immunohistochemical analysis of papillomavirus binding factor (PBF) expression in 20 primary tumor samples	Over-expression (grade+++) of PBF is correlated with poorer OS	*P* < 0.05
Kikuta et al. (2009)	Immunohistochemical analysis of nucleophosmin (NPM) expression in 34 primary tumor samples	Nuclear expression of NPM is correlated with poorer OS	*P* < 0.01
Scotlandi et al. (2009)	Quantitative RT-PCR analysis of membrane-bound microsomal glutathione *S*-transferase (*MGST1)* expression in 42 primary tumor samples	Low expression of *MGST1* is correlated with improved EFS	*P* = 0.02
Perbal et al. (2009)	Immunohistochemical analysis of CCN3 expression in 125 primary tumor samples	High expression (grade++ or higher) of CCN3 is correlated with poorer prognosis.	*P* = 0.05
Luo et al. (2009)	Immunofluorescent analysis of glutathione *S*-transferase mu 4 (GSTM4) expression in 44 primary tumor samples	High expression of GSTM4 is correlated with poorer OS	*P* = 0.054
Zambelli et al. (2010)	Immunohistochemical analysis of lectin galactoside-binding soluble 3 binding protein (LGALS3BP) expression in 274 primary tumors samples	Expression of LGALS3BP is correlated with improved EFS and OS	*P* = 0.04 and 0.03 respectively
Meynet et al. (2010)	Immunohistochemical analysis of Xg expression in 97 primary tumor samples	Expression of Xg is correlated with poorer EFS and OS	*P* = 0.02
Bennani-Baiti et al. (2010)	Quantitative RT-PCR analysis of *CXCR4* and *CXCR7* expression in 49 primary tumor samples	High expression of both *CXCR4* and *CXCR7* is correlated with poorer OS	*P* = 0.0161
Berghuis et al. (2011)	Immunohistochemical analysis of T-lymphocytic infiltration in 20 primary tumor samples	Increased tumor infiltration of CD8 + T-cells is correlated with improved OS	*P* = 0.05
Bui et al. (2011)	Immunohistochemical analysis of Connexin 43 (Cx43) expression in 36 primary tumor samples	Higher expression scores of Cx43 is correlated poorer OS	*P* = 0.002
Fujiwara et al. (2011)	Immunohistochemical analysis of macrophage infiltration in 41 primary tumor samples	High levels of macrophage infiltration ([ > 30 CD68 cells/high-power field) is correlated with poorer OS	*P* = 0.0046
Machado et al. (2012)	Immunohistochemical analysis of desmoplakin, phosphorylated glycogen synthase kinase 3b (pGSK3β), ZO-1, Snail, and CK8/18 in 415 primary tumor samples	Expression of desmoplakin or pGSK3β is correlated with improved PFS. Expression of ZO-1 or Snail is correlated with improved overall survival. Expression of CK8/18 is correlated with a poorer prognosis.	*P* = 0.0044 (desmoplakin), *P* = 0.026 (pGSK3β), *P* = 0.006 (ZO-1), *P* = < 0.0001 (Snail), *P* = 0.034 (CK8/18)
Nakatani et al. (2012)	Quantitative RT-PCR analysis of miR-34a in 49 primary tumor samples	High expression of miR-34a is correlated with improved EFS and OS	*P* = 0.0001 and 0.0005 respectively

## Targeted Agents for ES: The Need for Predictive Biomarkers

A number of biological targets and potentially promising novel agents have been identified for ES, many of which were discussed at the aforementioned ENCCA summit (Kovar et al., 2012). For the purpose of this discussion, we will focus on two proteins which have recently generated a great deal of interest as potential therapeutic targets in ES; the receptor tyrosine kinase Insulin Growth Factor Receptor 1 (IGF-1R) and Poly (ADP-ribose) polymerase 1 (PARP1). IGF-1R is highly expressed by ES cells, and many studies have demonstrated the importance of the IGF-1R pathway in ES tumor models (van Valen et al., 1992; Hofbauer et al., 1993; Scotlandi et al., 1996, 1998; Toretsky et al., 2001; Kolb et al., 2008). Clinical application of IGF-1R directed antibodies resulted in dramatic responses in a few patients with refractory disease (Olmos et al., 2010b). However, in several subsequent larger trials in unselected ES populations, response rates have been only about 10%, albeit in heavily pre-treated patients (Olmos et al., 2010a; Atzori et al., 2011; Juergens et al., 2011; Pappo et al., 2011; Malempati et al., 2012; Tap et al., 2012). Unfortunately, serial collections of tumor tissue following antibody therapy to evaluate its effect on downstream target proteins have been deemed to be both excessively invasive and expensive (Ho and Schwartz, 2011). Therefore, whether IGF-1R targeted therapy has failed to provide significant response rates due to a lack of intended biologic activity against the tumor remains unknown. Nevertheless, blood and serum samples from these studies have been collected, and may yet yield helpful information in terms of biomarkers for IGF-1R directed therapy. Furthermore, a phase 2 study of an IGF-1R directed antibody combined with chemotherapy is ongoing in patients with metastatic and refractory ES (NCT#00563680). The results of these studies are eagerly anticipated by this committee and by the sarcoma clinical and research communities as a whole.

The ability to predict whether a patient is likely to respond to a novel agent greatly increases the chance of success of a targeted therapy and fosters personalized medicine more generally. A striking example of the benefits of a predictive biomarker is the identification of the subset of patients with non-small cell lung cancer (NSCLC) patients who will respond to Epidermal Growth Factor Receptor (*EGFR*)-directed therapy. Activating mutations in the *EGFR* gene are detectable in only a small minority of NSCLC patients but it is these patients who selectively respond to *EGFR*-directed therapy (Saintigny and Burger, 2012). Similarly, activating mutations in *KIT* and *PDGFRA* genes in gastrointestinal stromal tumors are predictive for clinical responses to imatinib (Heinrich et al., 2003). Such a biomarker does not yet exist for IGF-1R directed therapy in ES, although recent studies have suggested that differential expression and activation of the insulin receptor and nuclear localization of phosphorylated IGF-1R may be useful predictors of treatment response (Garofalo et al., 2011; Asmane et al., 2012). These findings require validation in larger studies, and highlight some valuable missed opportunities from earlier trials.

The findings that only a small subset of patients with relapsed ES respond to IGF-1R targeted monotherapy serve as a sobering example of the critical need for predictive biomarkers in this disease. As trials investigating novel agents move forward, it is paramount that strategies that will permit evaluation of predictive biomarkers be simultaneously implemented. This will enable identification of patients who may preferentially benefit from such interventions in the future and allow for more selective inclusion and exclusion of patients in a manner that will lead to improved response rates. One potential treatment modality to emerge from recent pre-clinical investigations is PARP1 inhibition. PARP1 is a key enzyme involved in single-strand repair of DNA (Wang et al., 2012). In 1999, Soldatenkov et al. reported elevated PARP1 expression in ES, and regulation of PARP expression by ETS transcription factors (Soldatenkov et al., 1999). More recently, Brenner et al. (2012) demonstrated that ES fusion proteins interact with PARP1, and that *in vitro* and *in vivo* models of ES are highly sensitive to the PARP1 inhibitor Olaparib alone and in combination with the drug temozolomide. Moreover, in a drug screening of several hundred cancer cell lines a marked and selective susceptibility of ES cell lines to Olaparib was also discovered (Garnett et al., 2012). Based on these promising pre-clinical data, PARP1 inhibitors have already entered clinical trials in adults with ES (NCT#01583543). Since PARP1 inhibition has already been evaluated in numerous different adult-onset tumor types, a variety of potential biomarkers of DNA repair currently exist [i.e., γ-H2AX, poly(ADP-ribose)] and could be incorporated for evaluation in future pediatric trials (Wang and Weaver, 2011). Furthermore, assays are being developed to analyze the activity of PARP1 inhibitors in peripheral blood cells as a potential surrogate for tumor biopsies (Ji et al., 2011). This option would be especially appealing in pediatric patients, in whom practitioners may be reticent to perform tumor biopsies for exploratory biomarker studies. Due to the availability of PARP1 inhibitors in clinical trials for adult-onset cancers, it is possible that phase I trials for pediatric ES patients will be developed. As these protocols are conceptualized, comprehensive parallel testing of DNA repair markers must be included to test the validity of these assays as predictive biomarkers. Successful validation of a predictive biomarker in concert with clinical assessment of PARP1 inhibitor efficacy will ensure that the potential benefits of these agents are suitably investigated as expeditiously as possible.

## Conclusion

Numerous prognostic biomarker studies for ES have been published in recent years. Of particular interest and potentially high clinical relevance are studies of cell-cycle proteins, sub-clinical disease, and CNAs. All of these have demonstrated prognostic significance in numerous retrospective studies and now need to be validated prospectively in larger cohorts of equivalently treated patients. The challenges in identifying and validating clinically relevant biomarkers in ES highlight a significant hurdle for the individualization of therapy in any rare cancer. Prospective therapeutic trials with standardized treatments remain the optimum source of biologic material and clinical correlative information to drive successful biomarker identification. Since these trials can take years to complete it is essential that biomarker studies be meticulously designed and incorporated up front in therapeutic studies. It is imperative that these studies are designed vigilantly to maximize levels of evidence and ensure adherence to REMARK guidelines. In addition, biomarkers that can be tested and validated on blood or fixed tumor specimens will have the best chance of translation into routine clinical practice. As new agents are developed, predictive biomarkers will need to be developed to assess the benefit of these therapies and rationally design treatment stratification based on likelihood of response. The choice of technical platforms must also be carefully considered in trials involving rare diseases. Although characteristics such as sensitivity are important when choosing a methodology, issues such as availability, cost-effectiveness, and sample requirements are equally important. Rare cancers require the participation of multiple institutions, and it is imperative that samples from each site are similarly collected and processed. Cooperative groups can play a critical role to ensure that biomarker studies are carefully selected, rigorously designed and, whenever possible, incorporated into therapeutic studies.

## Conflict of Interest Statement

The authors declare that the research was conducted in the absence of any commercial or financial relationships that could be construed as a potential conflict of interest.
